# Transcutaneous electrical acupoint stimulation alleviates insomnia, negative emotions, and neurotransmitter imbalance in methamphetamine withdrawal: A randomized controlled trial

**DOI:** 10.1097/MD.0000000000043508

**Published:** 2025-08-01

**Authors:** Xiong Zhang, Chun-li You, Sheng-jie Zhang, Fang Zhang, Zu-anna Yi, Wei Ren, Jia Li

**Affiliations:** a College of Acumox and Tuina, Guizhou University of Traditional Chinese Medicine, Guiyang, China; b Teaching and Research Office of Traditional Chinese Medicine, Guizhou Nursing Vocational College, Guiyang, China; c Medical Department, Kangxin Hospital of Guizhou Province, Guiyang, China.

**Keywords:** insomnia, methamphetamine, negative emotion, neurotransmitters, transcutaneous electrical acupoint stimulation

## Abstract

**Background:**

This randomized controlled trial investigated the therapeutic effects of transcutaneous electrical acupoint stimulation (TEAS) on insomnia, negative emotions, and neurotransmitter levels in patients undergoing methamphetamine (METH) withdrawal.

**Methods:**

We enrolled 84 eligible patients from the Kangxin Hospital of Guizhou Province, China (August 2023–December 2024), randomly allocating them to TEAS (n = 42) or sham-TEAS (n = 42) groups. The patients were divided into a TEAS group of 42 cases and a sham-TEAS group of 42 cases according to the randomized, single-blind and control principle. The 2 groups patients were given TEAS or sham-TEAS treatment for 45 minutes after treated with sleep health education. Interventions described above in weekdays were given once a day for 4 weeks, with a total of 4 consecutive weeks. The Pittsburgh Sleep Quality Index (PSQI), insomnia severity index (ISI), METH withdrawal symptom score, Hamilton Anxiety Scale (HAMA), Hamilton Depression Scale (HAMD), serum levels of 5-hydroxytryptamine, noradrenaline, gamma-aminobutyric acid and dopamine of each group were compared before treatment, after treatment and in follow-up period.

**Results:**

Baseline characteristics were comparable between groups (both *P* > .05). After 4 weeks of treatment, the scores of PSQI, ISI, METH withdrawal symptom, HAMA, HAMD in the TEAS group were significantly lower than those in the sham-TEAS group and the difference was statistically significant (both *P* < .001), serum levels of 5-hydroxytryptamine, gamma-aminobutyric acid and dopamine in the TEAS group were significantly higher than those in the sham-TEAS group, and serum levels of noradrenaline in the TEAS group were significantly lower than those in the sham-TEAS group (both *P* < .001). In follow-up period, the scores of PSQI, ISI, METH withdrawal symptom, HAMA, HAMD in the TEAS group were significantly lower than those in the sham-TEAS group, and the difference was statistically significant (both *P* < .001).

**Conclusion:**

TEAS effectively improved insomnia, anxiety and depression after METH withdrawal, it is worth of clinical application and promotion while significantly affecting serum levels of neurotransmitters.

## 1. Introduction

METH is a synthetic drug with strong addictive properties. At present, the abuse of METH is especially serious. According to an epidemiological report released by the United Nations Office reports on Drugs and Crime, over 30 million people worldwide are addicted.^[1]^ Another epidemiological survey demonstrated that more than 57.6% of METH addicts may appear insomnia after withdrawal, at the same time these insomnia patients may appear anxiety, depression and other psychiatric symptoms during sleep disorders.^[2,3]^ These psychiatric symptoms in METH abstainers are closely associated with success rate of withdrawal. Many abstainers tend to relapse METH in order to alleviate these psychiatric symptoms, which greatly compromises the efficiency of withdrawal. Relapse behavior may greatly harm the abstainers with METH and their families, including inducing crimes that can severely endanger public safety and cause large social and economic losses.

Obviously, the effective treatment of psychiatric symptoms after METH withdrawal has become a pressing issue in clinical practice. The most commonly used clinical treatment method is mainly drug therapy, pharmacological treatments relieve psychiatric symptoms through oral or injectable administration of antipsychotics, for example, METH abstainers with insomnia symptoms are given dexzopiclone, and those with depression symptoms are given paroxetine, while those with anxiety symptoms are treated with clonazepam. Actually, it is not ideal with single symptomatic treatment of antipsychotics, these antipsychotics effectiveness is controversial, and the use of antipsychotics is often accompanied by dependence of treat drug and gastrointestinal adverse reactions.^[4]^ Therefore, most of the METH abstainers prefer alternative therapies with fewer side effects and greater safety clinically. On the other hand, cognitive behavioral therapy and relaxation therapy have been used as complementary therapies of psychiatric symptoms after METH withdrawal have not yet been widely applied in clinical practice because of they have limited efficacy and are expensive.

Nowadays, acupuncture therapy guided by theory of traditional Chinese medicine are widely used across drug Addiction Treatment Center in China and even many other parts of Asia. Some clinical trials have demonstrated the effect of acupuncture therapy in treating psychiatric symptoms in abstainers with METH.^[5–7]^ However, there are problems such as lack of standardization in the quantity of stimulus and traumatic skin injury with the use of acupuncture, the application of acupuncture therapy sometimes is limited. Transcutaneous electrical acupoint stimulation (TEAS) is a new acupoint stimulation treatment, generate electronic stimuli of a certain frequency on acupoints or skin to induce the physiological effects in the patient’s body based on the methods of traditional acupuncture, which could activate the physiological mechanisms of self-adjustment.^[8–10]^ It combines the advantages of acupuncture with electrical stimulation, thus may obtain more significant efficacy than single acupuncture. Therefore, this study aims to evaluate the efficacy of TEAS versus sham-TEAS in improving insomnia, anxiety, and depression symptoms in METH abstainers, investigate its effects on serum neurotransmitter levels (5-hydroxytryptamine [5-HT], noradrenaline [NE], gamma-aminobutyric acid [GABA], dopamine [DA]), and establish TEAS as a noninvasive alternative therapy for the psychiatric symptoms METH after withdrawal.

## 2. Materials and methods

### 2.1. Research participants

**Figure 1.**
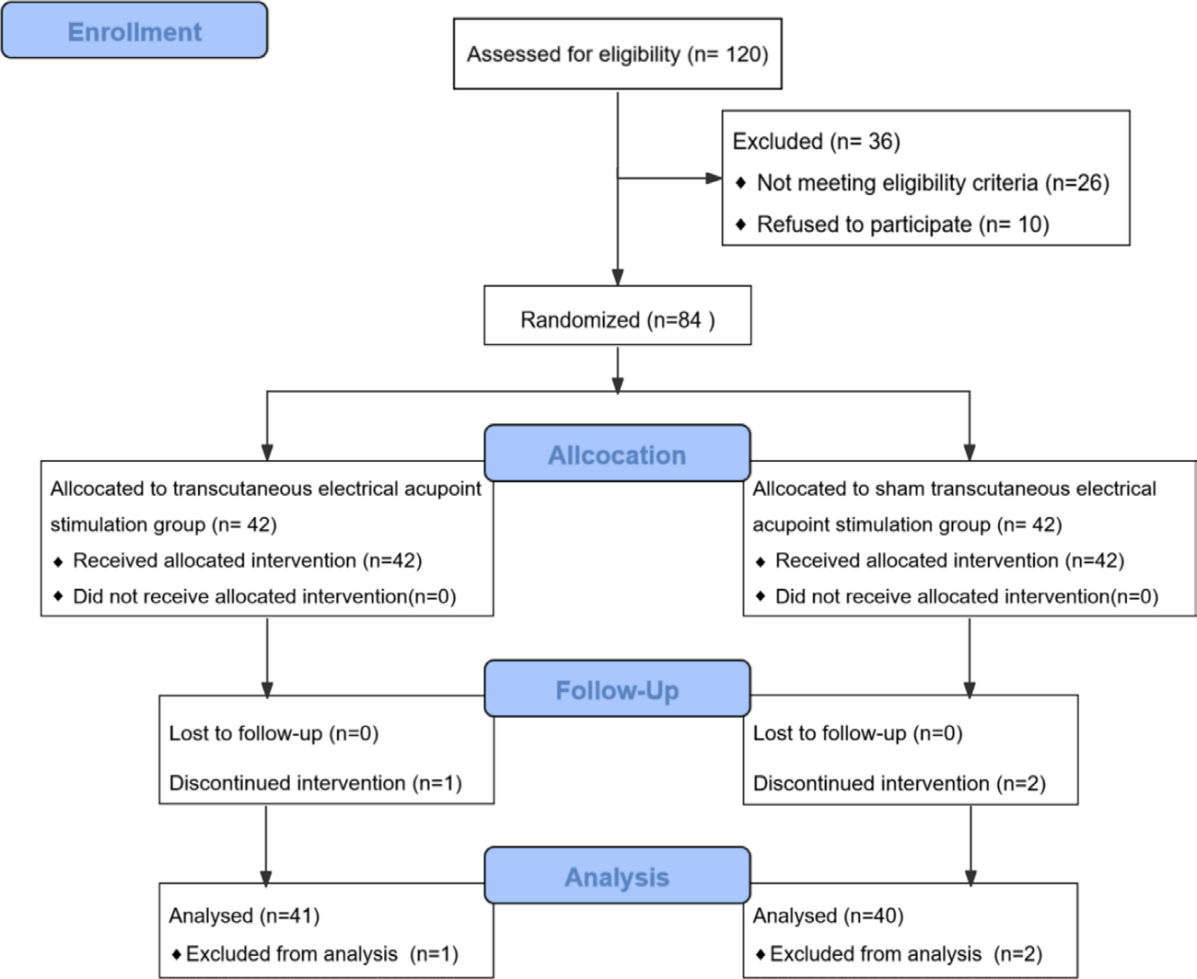
CONSORT flow diagram of the participants.

Participants were consecutively screened by 2 independent clinicians blinded to group allocation. Randomization was stratified by baseline Pittsburgh Sleep Quality Index (PSQI) scores to avoid potential biases. A total of 120 METH abstainers at the Kangxin Hospital of Guizhou Province in China were enrolled in this study from August 2023 to December 2024. After 26 did not meet the inclusion criteria and 10 refused to participate, 84 eligible patients were selected from the hospital. The patients were divided into 2 groups. One is the TEAS group of 42 cases and the other is the sham-TEAS group of 42 cases according to the randomized, single-blind and control principle (Fig. 1). The study was approved by the Institutional Review Board of our hospital (No. GZKFJDYY20230601). All patients signed an informed consent form before the trial.

### 2.2. Inclusion and exclusion criteria

Inclusion criteria: More than 12 months of MEHT abuse and more than 6 months of dependence history. Adults aged 18 to 60 years old. METH negative in urinalysis, and receiving natural withdrawal therapy for ≥12 months. The Pittsburgh Sleep Quality Index score was more than 7 points. The Hamilton Anxiety Scale score was more than 8 points. The Hamilton’s Depression Scale score was more than 8 points. Able to cooperate with the study, not received other treatments previous 2 months. Patients who have signed an informed consent form.

Exclusion criteria: Patients who having adverse reaction to the electrical stimulation used in this experiment or serious trauma in the skin area of the acupoint that was not healed. Patients who having serious diseases of the heart, liver, lung, kidney, and other organs. Patients with psychiatric history. Women during lactation or pregnancy. Patients with incomplete case data.

Rejection and shedding criteria: Patients who terminated the treatment due to electrical stimulation fainting and other adverse reactions. Patients who cannot continue treatment due to external factors.

### 2.3. Intervention

**Figure 2.**
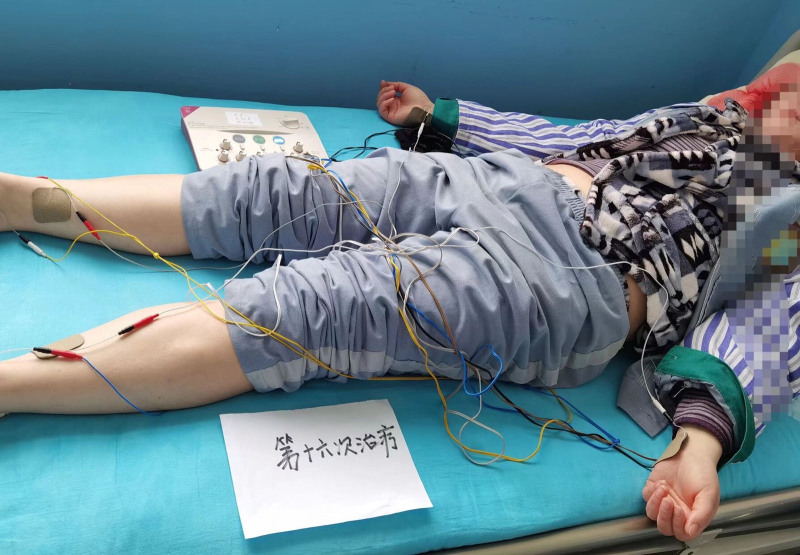
Diagram of the TEAS treatment for participants. TEAS = transcutaneous electrical acupoint stimulation.

The TEAS group received transcutaneous electrical acupoint stimulation treatment for 45 minutes at Baihui (GV20), Yintang (DU29), and bilateral Nei guan (PC6), Shen men (HT7), Zu san li (ST36), San yin jiao (SP6) and Anmian (Ex-HN18) with astimulator (model: SDZ-1l, purchased from: Suzhou Medical Appliances Co. Ltd., Suzhou, China). These acupoints are based on traditional anatomical positions. Prior to each treatment, calibration was performed with the stimulator, acupoints were routinely disinfected with 75% alcohol, electrode tabs were placed on the skin of these acupoints. We applied dilatational waves (2/100 Hz frequency) with current intensity titrated to patient tolerance (5–15 mA range). The sham-TEAS group was only connected to the electrodes without initiating electrical stimulation. Patients were given intervention treatment once a day every weekday for 4 continuous weeks. The total treatment times is 20. Meanwhile, patients of both groups were given a 1-hour sleep health education before every intervention treatment. If adverse reactions appeared during the treatment period, treatment should be terminated immediately (Fig. 2).

### 2.4. Outcome measures and evaluation criteria

All outcome measures was assessed before and after treatment, follow-up period (2 weeks after the end of treatment) among all groups. The changes of overall sleep quality after METH withdrawal were assessed according to the PSQI scale scores in both groups. The total scores of PSQI scale is calculated by totaling all the component scores including sleep quality, sleep latency, sleep duration, sleep efficiency, sleep disturbances, and daytime dysfunction, the total scores ranged from 0 to 21, lower scores denote better sleep quality. The insomnia symptoms was assessed with the insomnia severity index (ISI) scale scores in both groups, the total scores ranged from 0 to 28, higher scores denote increasingly severe insomnia symptoms. The anxiety symptoms was assessed with the HAMA scale in both groups, it consisted of 2 subscales, including psychic anxiety and somatic anxiety, in which the symptoms were assessed using 5 grades, where grade 0 to grade 4 referred to no symptom to extremely severe symptoms. The depression symptoms was assessed with the HAMD scale, It consisted of 7 subscales, including anxiety/somatization, weight, cognitive impairment, diurnal variation, blockage, sleep disorders, and hopelessness, in which the symptoms were assessed using 5 grades, where grade 0 to grade 4 referred to no symptom to extremely severe symptoms. The withdrawal symptoms was assessed with the METH withdrawal symptoms scale^[11]^ in both groups, it consisted of 14 questions, resulting in a total scores ranging from 0 to 42, higher METH withdrawal symptoms scale scores denote increasingly severe withdrawal symptoms. In addition, the serum levels of neurotransmitters was assessed with the ELISA in both groups, the change reaction of neurotransmitters in the serum can be reflected, including 5-TH, GABA, DA, and NE.

### 2.5. Statistical analysis

Before the start of the study, computational formula of sample size was used to calculate the sample size required for the present study and referred to the data reported in previous literatures. The sample size estimation formula was n_1_ = n_2_ = 2[(μ_*α*_+μ_β_)/(δ/*σ*)]^2^ + 1/4μ_α_^2^, where n_1_ and n_2_ were the required sample size for the 2 groups, μ_α_ and μ_β_ were the μ values corresponding to the test level α and the subtype error probability β, δ was the difference between the 2 groups of sample means, σ was the population standard deviation. Referred to the data reported in previous literatures predicted δ/σ = 0.79, specified α = 0.05, β = 0.1, μ_α_ = 1.645, μ_β_ = 1.282, calculated n_1_ = n_2_ = 35, considering 20% loss rate, so each group sample was extended to 42 cases, and a total of 84 patients were needed. SPSS 26.0 statistical software was used for data processing. Continuous variables (e.g., PSQI scores, neurotransmitter levels) were expressed as mean ± standard deviation ($\bar{x}\pm s$) and compared between groups using independent samples *t* tests (normal distribution) or Mann–Whitney *U* tests (non-normal distribution), while categorical variables (e.g., gender) were analyzed with chi-square tests. Within-group pre-post changes were assessed via paired *t* tests or Wilcoxon signed-rank tests. Normality was verified using the Shapiro–Wilk test. A 2-tailed *P* < .05 defined statistical significance, with Bonferroni correction applied for multiple comparisons. Missing data were addressed via per-protocol analysis (n = 81), and sensitivity analyses confirmed robustness. The chosen tests ensured appropriate evaluation of group differences and treatment effects while controlling for Type I/II errors.

## 3. Results

### 3.1. Baseline characteristics data

A total of 84 patients who were accordance with the inclusion criteria and signed informed consent were enrolled. Among them, there were 3 patients terminated the treatment, 2 referral patients due to external factors, and 1 adverse reactions patients, which resulted in a total of 81 patients who actually completed the study (41 cases of the TEAS group and 40 cases of the sham-TEAS group). No significant differences existed in baseline characteristics data, including gender, age, METH abuse time, average daily METH use, and METH withdrawal time between the TEAS and sham-TEAS groups (both *P* > .05, Table 1).

**Table 1:** Comparison of baseline characteristics data between the each groups.

	Sham-TEAS group (n = 40)	TEAS group (n = 41)	*x*^2^/*t*/z value	*P* value
Gender (male/female)	22/18	21/20	0.116	.733
Age (yr)	28.80 ± 6.31	29.85 ± 6.96	−0.713	.478
METH abuse time (yr)	5.95 ± 2.71	6.73 ± 2.70	−1.250	.211
Average daily METH use (g)	2.83 ± 0.70	2.80 ± 0.72	−0.261	.794
METH withdrawal time (mo)	12.80 ± 0.85	12.98 ± 1.15	−0.354	.724

METH = methamphetamine, TEAS = transcutaneous acupoint electrical stimulation.

### 3.2. Comparison of the overall sleep quality and insomnia symptoms between 2 groups

There was no significant difference in total scores and each component scores of the PSQI scale between the TEAS group and the sham-TEAS group before treatment (both *P* > .05, Tables 2 and 3). The total scores and each component scores of the PSQI scale in the TEAS group were significantly lower than the scores tested in the sham-TEAS group after treatment and in follow-up period, and the difference was statistically significant (both *P* < .001, Tables 2 and 3).

**Table 2:** Comparison of the total scores of PSQI scale and ISI scale between the each groups.

	PSQI total scores			ISI total scores		
	Sham-TEAS group (n = 40)	TEAS group (n = 41)	*P*	Sham-TEAS group (n = 40)	TEAS group (n = 41)	*P*
Before treatment	14.65 ± 1.37	14.34 ± 1.20	.322	18.10 ± 3.81	18.22 ± 3.37	.475
After treatment	14.05 ± 1.22***	4.10 ± 1.45	<.001	17.60 ± 1.79***	11.76 ± 2.84	<.001
Follow-up period	14.68 ± 1.12***	4.12 ± 1.31	<.001	18.50 ± 2.26***	10.07 ± 2.15	<.001

ISI = insomnia severity index, PSQI = Pittsburgh Sleep Quality Index.

*** Compared with sham-TEAS group, *P* < .001.

**Table 3:** Comparison of the score of PSQI each component between the each groups.

	Sleep quality			Sleep latency			Sleep duration		
	Sham-TEAS group (n = 40)	TEAS group (n = 41)	*P*	Sham-TEAS group (n = 40)	TEAS group (n = 41)	*P*	Sham-TEAS group (n = 40)	TEAS group (n = 41)	*P*
Before treatment	2.43 ± 0.50	2.49 ± 0.51	.573	2.68 ± 0.47	2.54 ± 0.60	.332	2.48 ± 0.51	2.46 ± 0.50	.917
After treatment	2.53 ± 0.51***	0.76 ± 0.58	<.001	2.60 ± 0.50***	0.73 ± 0.45	<.001	2.30 ± 0.46***	0.63 ± 0.49	<.001
Follow-up period	2.30 ± 0.46***	0.71 ± 0.51	<.001	2.25 ± 0.59***	0.73 ± 0.45	<.001	2.43 ± 0.50***	0.66 ± 0.53	<.001

	Sleep efficiency			Sleep disturbances			Daytime dysfunction		
	Sham-TEAS group (n = 40)	TEAS group (n = 41)	*P*	Sham-TEAS group (n = 40)	TEAS group (n = 41)	*P*	Sham-TEAS group (n = 40)	TEAS group (n = 41)	*P*
Before treatment	2.05 ± 0.55	1.95 ± 0.55	.417	2.45 ± 0.50	2.41 ± 0.50	.750	2.58 ± 0.50	2.49 ± 0.51	.435
After treatment	2.00 ± 0.51***	0.61 ± 0.49	<.001	2.20 ± 0.41***	0.71 ± 0.46	<.001	2.43 ± 0.50***	0.66 ± 0.48***	<.001
Follow- up period	2.60 ± 0.55***	0.63 ± 0.49^*^	<.001	2.53 ± 0.51***	0.76 ± 0.49^*^	<.001	2.58 ± 0.50***	0.63 ± 0.49***	<.001

*** Compared with sham-TEAS group, *P* < .001.

There was no significant difference in the total scores of the ISI scale between the TEAS group and the sham-TEAS group before treatment (*P* > .05, Table 2). The total scores of the ISI scale in the TEAS group were significantly lower than the scores tested in the sham-TEAS group after treatment and in follow-up period, and the difference was statistically significant (both *P* < .001, Table 2).

### 3.3. Comparison of the anxiety symptoms between 2 groups

There was no significant difference in total scores, somatic and psychic anxiety scores of the HAMA scale between the TEAS group and the sham-TEAS group before treatment (both *P* > .05, Table 4). The total scores, somatic and psychic anxiety scores of the HAMA scale in the TEAS group were significantly lower than the scores tested in the sham-TEAS group after treatment and in follow-up period, and the difference was statistically significant (both *P* < .001, Table 4).

**Table 4:** Comparison of the scores of the HAMA scale between the each groups.

	Total HAMA score			Somatic anxiety score			Psychic anxiety score		
	Sham-TEAS group (n = 40)	TEAS group (n = 41)	*P*	Sham-TEAS group (n = 40)	TEAS group (n = 41)	*P*	Sham-TEAS group (n = 40)	TEAS group (n = 41)	*P*
Before treatment	23.58 ± 2.32	22.76 ± 3.14	.385	11.65 ± 1.56	11.37 ± 1.58	.337	11.93 ± 1.44	11.39 ± 1.83	.294
After treatment	24.48 ± 1.30***	6.41 ± 1.32	<.001	12.50 ± 0.51***	3.27 ± 1.07	<.001	11.98 ± 1.14***	3.15 ± 0.96	<.001
Follow-up period	24.55 ± 0.90***	5.93 ± 1.39	<.001	12.23 ± 0.70***	3.07 ± 0.98	<.001	12.33 ± 0.73***	2.85 ± 0.96	<.001

HAMA = Hamilton Anxiety Scale.

*** Compared with sham-TEAS group, *P* < .001.

### 3.4. Comparison of the depression symptoms between 2 groups

There was no significant difference in the total scores of the HAND scale between the TEAS group and the sham-TEAS group before treatment (*P* > .05). The HAND scores tested after treatment and the scores tested within the follow-up period in the TEAS group were significantly lower than the scores of sham-TEAS group (both *P* < .001, Table 5).

**Table 5:** Comparison of the scores of the HAMD scale between the each groups.

	Sham-TEAS group (n = 40)	TEAS group (n = 41)	*P*
Before treatment	29.23 ± 2.95	29.63 ± 2.99	.537
After treatment	30.08 ± 2.68***	13.83 ± 1.92	<.001
Follow-up period	29.10 ± 2.27***	12.12 ± 1.54	<.001

HAMD = Hamilton Depression Scale.

*** Compared with sham-TEAS group, *P* < .001.

### 3.5. Comparison of the METH withdrawal symptoms between 2 groups

There was no significant difference in the total scores of the METH withdrawal symptoms scale between the TEAS group and the sham-TEAS group before treatment (*P* > .05). The total scores of the METH withdrawal symptoms scale tested after treatment and the scores tested within the follow-up period in the TEAS group were significantly lower than the scores in the sham-TEAS group, and the difference was statistically significant (both *P* < .001, Table 6).

**Table 6:** Comparison of the scores of the METH withdrawal symptoms scale between the each groups.

	Sham-TEAS group (n = 40)	TEAS group (n = 41)	*P*
Before treatment	24.25 ± 4.33	24.71 ± 3.94	.620
After treatment	24.58 ± 4.22***	12.05 ± 3.41	<.001
Follow-up period	25.43 ± 4.44***	10.44 ± 2.21	<.001

*** Compared with sham-TEAS group, *P* < .001.

### 3.6. Comparison of the serum levels of neurotransmitters between 2 groups

The results showed that there was no statistical difference in the serum levels of 5-HT, DA, GABA, NE between the 2 groups before treatment (both *P* > .05, Table 7). After 4 weeks of treatment, the serum levels of 5-HT, GABA, and DA in the TEAS group were significantly higher than those in the sham-TEAS group, and serum levels of NE in the TEAS group were significantly lower than those in the sham-TEAS group (both *P* < .001, Table 7).

**Table 7:** Comparison of the serum levels of neurotransmitters between the each groups.

Serum levels of neurotransmitters		Sham-TEAS group (n = 40)	TEAS group (n = 41)	*P*
5-HT (ng/mL)	Before treatment	21.55 ± 4.58	20.55 ± 4.30	.313
	After treatment	19.65 ± 4.60***	29.90 ± 4.83	<.001
GABA (μmol/L)	Before treatment	13.86 ± 3.09	13.51 ± 3.26	.688
	After treatment	12.93 ± 2.37***	19.23 ± 2.95	<.001
DA (ng/mL)	Before treatment	8.04 ± 1.50	8.47 ± 1.40	.190
	After treatment	7.61 ± 1.28***	11.58 ± 1.93	<.001
NE (μmol/L)	Before treatment	5.37 ± 1.04	5.28 ± 1.02	.716
	After treatment	5.28 ± 1.11***	3.68 ± 1.07	<.001

5-HT = 5-hydroxytryptamine, DA = dopamine, GABA = gamma-aminobutyric acid, NE = noradrenaline.

*** Compared with sham-TEAS group, *P* < .001.

## 4. Discussions

METH is different from other opioid addiction drugs for its neurovirulence property, which leads to long-term psychotic symptoms after withdrawal, including insomnia, anxiety, and depression. Many abstainers tend to relapse on METH to alleviate these psychotic symptoms, a behavior that greatly compromises their withdrawal success. This underscores the need for effective interventions targeting psychiatric symptoms after METH withdrawal. While pharmacological treatments (such as dexzopiclone for insomnia, paroxetine for depression) are commonly used, their safety remains controversial due to side effects like dependence and gastrointestinal distress.

Acupuncture is an external treatment of traditional Chinese medicine, wherein filiform needles are inserted subcutaneously at acupoints or lesions to stimulate the patient’s body to engage in disease prevention and control.^[12,13]^ As a kind of broad-spectrum medical technique, it is effective for the treatment of psychiatric symptoms after withdrawal METH has been corroborated.^[14–17]^ Acupuncture is widely used in China other parts of world. However, it should be widely acknowledged that the traumatic of acupuncture therapy is always associated with adverse reactions and controversial safety. Therefore, exploring safer noninvasive therapies for treatment of the psychiatric symptoms after METH withdrawal will be increasingly crucial.

By comparison, TEAS combines the advantages of both acupuncture and electrical stimulation, while providing noninvasive yet effective stimulation to acupoints. Our results support this advantage, the baseline period data in 2 groups patients had no significant difference. After TEAS treatment, the scores of PSQI scale and ISI scale decreased more in the TEAS group than the sham-TEAS group, this suggests that the noninvasive electrical stimulation in the TEAS therapy can obviously improve the overall sleep quality and alleviate insomnia symptom after METH withdrawal. In terms of the negative emotions, the total scores, somatic and psychic anxiety scores of the HAMA scale and the total scores of the HAND scale of the TEAS group was significantly lower than that of the sham-TEAS group after receiving 4 weeks of treatment, mirroring outcomes reported by study on EA treatment for negative emotions after METH withdrawal.^[5]^ In terms of the METH withdrawal symptom, we selected the modified METH withdrawal symptoms scale according to the measurement developed by Beijing University to evaluate the patients. After TEAS treatment, patients with the total scores of the METH withdrawal symptoms scale decreased in the TEAS group, compared with the sham-TEAS group. This suggests that the TEAS therapy can obviously improve patients with the METH withdrawal symptoms, and effectively inhibit relapse of METH abstainers, aligning with previous similar study, who reported therapeutic effects with electro-acupuncture on psychiatric symptoms after withdrawal METH.^[7]^ In addition, all patients were followed up 2 weeks after the end of treatment. The study found that stopping the TEAS therapy did not lead to a rebound of psychiatric symptoms in most of the patients who entered the follow-up period.

Evidence from clinical studies has indicated that the level of monoamine neurotransmitters was associated with pathomechanism of psychiatric symptoms,^[18–20]^ especially it is closely related to the pathomechanism of psychiatric symptoms after METH withdrawal.^[21,22]^ Several studies have shown that the acupuncture precisely can have benign regulation function of 2-way, which can adjust the level of the 5-TH, DA, GABA, NE, and other monoamine neurotransmitters in the body, thereby to balance physiological functions between nervous, endocrine and immune systems, and thus improve insomnia, anxiety, depression and other psychiatric symptoms after METH withdrawal.^[23–25]^ Hence, we hypothesize that the TEAS therapy may be used as an ideal alternative therapy, and analyzed the effect of noninvasive electrical stimulation in the serum level of monoamine neurotransmitters. In our study, the ELASA analysis showed there was no statistical difference in the serum levels of these neurotransmitters between the 2 groups before treatment. After 4 weeks treatment, the serum levels of 5-HT, GABA, and DA in the TEAS group were significantly higher than those in the sham-TEAS group, This suggests that our results and the previously reported of traditional acupuncture on psychiatric symptoms after withdrawal METH conclusions almost the same,^[16]^ but prior work show that the serum levels of 5-HT, GABA were significantly reduce.^[6]^ However, our study uniquely documents sustained effects of TEAS on NE levels, a finding not previously reported.

The synergistic effect of acupoints combined forms the therapeutic basis of TEAS therapy, Thus the choice of acupoints would be crucial for the effects of TEAS treat diseases. THE traditional Chinese medicine theory posits that Qi and blood is the material basis of mental activities in the body, the unhindered circulation of Qi and blood along the meridians is vital for mental activities.^[26,27]^ “Blood stasis” and “Qi stagnation” is the primary pathological product after METH withdrawal can blocking the meridians and affect the normal flow of Qi and blood in the meridians, resulting in negative emotions such as anxiety, depression and insomnia.^[28]^ Stimulating on acupoints of each meridians can regulate Qi and blood of the body and clear the spirits because the acupoints is main reflecting area where the Qi and blood runs on the meridians.^[29,30]^ Nei guan (PC6) and Shen men (HT7) can regulate the mind, promote sleep by combination with Zu san li (ST36), San yin jiao (SP6) and Anmian (Ex-HN18). Meanwhile, stimulations on acupoints of the Governor Vessel regulate spirit of the body because the head is the main area where the Governor Vessel runs.^[31,32]^ Thus, the acupoints of the Governor Vessel is specifically applied to treating psychiatric diseases, according to the guidance of the theory, the acupoints of the Governor Vessel including Baihui (GV20), Yintang (DU29) were selected, and the TEAS was performed for 4 weeks.

In summary, the TEAS significantly improves psychiatric symptoms after METH withdrawal, likely via neurotransmitter modulation, with its noninvasiveness and durability supporting clinical adoption. However, limitations such as small sample size, brief follow-up period, and absence of an active comparator necessitate future larger-scale, longer-term, and mechanism-focused studies.

## Acknowledgments

Thanks to all the staffs of the Kangxin Hospital of Guizhou Province for their support and assistance to this study.

## Author contributions

**Conceptualization:** Xiong Zhang, Chun-li You.

**Data curation:** Xiong Zhang, Chun-li You, Sheng-jie Zhang, Zu-anna Yi.

**Formal analysis:** Xiong Zhang, Chun-li You, Sheng-jie Zhang, Fang Zhang, Wei Ren.

**Funding acquisition:** Xiong Zhang, Jia Li, Chun-li You.

**Project administration:** Xiong Zhang, Jia Li.

**Writing – original draft:** Xiong Zhang, Chun-li You.

**Writing – review & editing:** Xiong Zhang, Chun-li You, Fang Zhang, Jia Li.
